# Dynamic Mechanical Loading Modulates Extracellular Vesicle Production and Cargo in Meniscus Fibrochondrocytes

**DOI:** 10.1002/admi.202600012

**Published:** 2026-07-12

**Authors:** Catherine Cheung, Zizhao Li, Sung-Han Jo, Se-Hwan Lee, Il Keun Kwon, Jina Ko, Su Chin Heo

**Affiliations:** 1 McKay Orthopaedic Research Laboratory, Department of Orthopaedic Surgery, University of Pennsylvania, Philadelphia, Pennsylvania, USA; 2 Department of Bioengineering, University of Pennsylvania, Philadelphia, Pennsylvania, USA; 3 Department of Dentistry, College of Dentistry, Kyung Hee University, Seoul, Republic of Korea; 4 Department of Pathology and Laboratory Medicine, Perelman School of Medicine, University of Pennsylvania, Philadelphia, Pennsylvania, USA

**Keywords:** extracellular vesicles, mechanical loading, meniscus, regenerative medicine

## Abstract

The meniscus is a fibrocartilaginous tissue critical for knee stability and load distribution, but its poor healing capacity makes regeneration after injury a major clinical challenge. Extracellular vesicles (EVs) are emerging as promising cell-free therapeutics for tissue regeneration. However, their clinical translation remains limited by low yield and variable bioactivity. This study investigates how dynamic mechanical loading regulates the production, composition, and function of meniscus fibrochondrocyte-derived EVs (MFC-EVs). Using a custom bioreactor system, cells are subjected to physiologically relevant cyclic tensile loading. Mechanical stimulation significantly increases EV production, likely through the ESCRT-independent pathway, without altering vesicle size or tetraspanin expression. “Mechanically primed” EVs enhance aggrecan expression in recipient mesenchymal stromal cells (MSCs), mirroring the loading-response phenotype of their source cells. Proteomic profiling identifies 380 unique proteins across all EV groups, with primed EVs enriched in extracellular matrix- and cytoskeleton-associated proteins, along with pathways related to tissue morphogenesis, cell migration, and cartilage development. Together, these findings show that physiologic mechanical loading enhances MFC-EV yield and is associated with distinct EV cargo profiles and recipient MSC gene expression responses. This work provides a scalable, biologically inspired strategy for engineering EV-based approaches with potential applications in meniscus repair and musculoskeletal regeneration.

## 1 | Introduction

The menisci are fibrocartilaginous tissues that provide joint stability, shock absorption, and load distribution in the knee joints [1]. Despite their important role in knee biomechanics, the menisci are highly susceptible to injury due to the complex and repetitive loading environment of the joint. Current nonsurgical treatments such as physical therapy and/or corticosteroid injections primarily aim to alleviate pain rather than promote tissue repair [1, 2]. Surgical intervention is typically offered when conservative treatments fail or when patients present with mechanical symptoms such as catching or locking of the knee [1, 2]. Although tears localized in the outer one-third of the meniscus, the vascular “red” zone, may heal following repair, the majority of meniscus injuries occur in the avascular inner “white” region and are treated by arthroscopic partial meniscectomy (APM), in which the damaged portion of tissue is excised. While APM can effectively relieve symptoms, it fails to restore the native biomechanical or biological functions of the meniscus [3, 4]. Consequently, approximately two-thirds of patients who undergo meniscus tear treatment develop osteoarthritis (OA) within 5–15 years after injury, regardless of intervention [5]. Given that OA is a leading cause of disability worldwide, affecting nearly 600 million individuals globally and projected to increase with aging populations [6], developing effective regenerative strategies that prevent OA onset by effectively repairing meniscal injuries remains an urgent clinical need.

The meniscus has a particularly limited healing response largely due to its minimal vascularity and dense extracellular matrix (ECM), which restricts immune cell infiltration, progenitor cell recruitment, and nutrient diffusion [7, 8]. Recently, extracellular vesicles (EVs) have emerged as a promising strategy to address these limitations. EVs are nanoscale, membrane-bound vesicles that transport bioactive cargo, including nucleic acids, lipids, proteins, and metabolites, that reflect the physiological state of their cell sources [9, 10]. Through paracrine and autocrine signaling, EVs regulate a wide range of biological processes, including cell proliferation [11, 12], cell migration [13], differentiation [14], matrix remodeling [15], angiogenesis [16, 17], and immune modulation [18, 19]. Their small size enables diffusion through dense matrices such as those of cartilage or meniscus. Importantly, compared to direct cell transplantation, EVs have greater stability and lower immunogenicity [9, 20, 21], positioning them as attractive candidates for next-generation, cell-free regenerative therapeutics. However, the clinical translation of EV-based therapies remains limited by variability in cargo composition and function, which depend on the type and physiological state of their cell source. Moreover, conventional production methods yield low quantities, requiring large-scale cell expansion to achieve therapeutic doses. These challenges highlight the need for strategies that enhance both the yield and bioactivity of EVs for regenerative therapeutics.

In musculoskeletal applications, mesenchymal stromal cell (MSC)-derived EVs have demonstrated therapeutic potential by alleviating inflammation through macrophage repolarization, promoting cartilage matrix synthesis, and delaying OA progression in preclinical models [11, 22, 23]. Similarly, chondrocyte-derived EVs enhance chondrogenesis in MSC-based engineered cartilage constructs [24]. Despite these advances, little is known about EVs secreted by meniscus fibrochondrocytes (MFCs), the primary resident cells of the meniscus. MFC-derived EVs may mediate intra-meniscal communication and crosstalk with surrounding progenitor cells; however, their production, regulation, and function remain largely unexplored.

The knee joint is a highly dynamic, mechanically active environment that supports weightbearing and mobility. Mechanical stimulation is essential not only for joint development but also for maintaining tissue homeostasis in adulthood [25, 26]. Clinically, controlled mechanical loading, such as that achieved through physical therapy, often can yield outcomes comparable to surgical intervention [27–30]. However, the underlying cellular and molecular mechanisms remain poorly defined. Given that EVs are central mediators of intercellular communication [9, 10], they may serve as the key effectors coupling mechanical cues to homeostatic and regenerative responses in the meniscus.

Therefore, in this study, we investigated how dynamic mechanical loading influences MFC-EV production and the subsequent effects of these EVs on progenitor cell (e.g., MSC) behavior. To do this, we used a custom bioreactor system to apply cyclic tensile loading to MFC-laden aligned polycaprolactone (PCL) nanofibrous scaffolds, and the secreted EVs were collected from the conditioned media. Mechanical loading significantly increased EV yield without altering their morphology or surface markers. Furthermore, MSCs treated with these “mechanically primed” MFC-EVs displayed gene expression changes, most notably enhanced aggrecan expression, that mirrored the EV cell source, indicating load-dependent signal transmission between cells. Together, these findings identify mechanically primed MFC-EVs as likely mediators of matrix regulation, suggesting their potential to be a cell-free therapeutic strategy for meniscus repair. This work further indicates the potential of integrating mechanical cues with EV-based approaches to advance biologically inspired therapies for joint regeneration.

## 2 | Results and Discussion

### 2.1 | Dynamic Mechanical Loading Enhances EV Secretion by Meniscus Cells

The meniscus resides in a dynamically active joint, where physiological mechanical loading is essential for maintaining tissue integrity and function. Both overloading and underloading disrupt this delicate balance, resulting in matrix degradation and loss of homeostasis [31, 32]. In this context, we sought to determine whether physiologically relevant tensile loading [33] modulates MFC-EV secretion and function. Given that meniscus cells are continuously exposed to mechanical forces during daily activities and that these forces regulate cell behavior and differentiation [34], we first investigated whether dynamic loading influences EV secretion by outer zone MFCs and bone marrow-derived MSCs cultured on aligned PCL nanofibrous scaffolds (Figure 1a). Aligned PCL nanofibrous scaffolds were used to mimic the native anisotropic matrix of the outer meniscus, where collagen fibers are predominantly aligned circumferentially in response to tensile hoop stresses [1]. Dynamic tensile loading (L) of MFCs increased their EV yield by five- to tenfold compared to static free-swelling non-loading (NL) culture conditions across all experiments (Figure 1b). To determine whether this effect would be cell type-specific, we applied the same loading regimen to MSCs and observed a four- to sixfold amplification of EV secretion relative to NL controls (Figure 1c). These results demonstrate that dynamic mechanical stimulation broadly promotes EV secretion in multiple cell types in the knee joint. These findings are consistent with prior reports showing that EV biogenesis and release are mechanosensitive processes influenced by cues including substrate stiffness, shear stress, and mechanical loading [35–37]. Consistent with this, cyclic tensile loading has been reported to increase EV yield of C2C12 muscle progenitor cells [14], periodontal ligament cells [38], and bone marrow-derived MSCs [39]. Other approaches, such as fluid shear stress and hypoxia, have also been used to enhance EV yield in endothelial cells [40] and MSCs [41], respectively. Compared to these methods, cyclic tensile loading provides a physiologically relevant and controllable stimulus, especially when applied to outer meniscus MFCs cultured on aligned scaffolds. While the degree of EV enhancement depends on cell type and experimental conditions, our results suggest that cyclic tensile loading is an effective approach for increasing EV production in meniscus-derived cells. In addition, this bioreactor-based approach offers a scalable method to boost EV production. Combining mechanical loading with other stimuli may further improve EV yield and function, although this will need to be tested in future studies.

As the primary focus of this study was on MFC-EVs, all subsequent analyses were performed using MFC-EVs. To confirm EV identity and assess morphological characteristics, we next performed multiple EV characterizations. Nanoparticle tracking analysis (NTA) revealed comparable size distributions between static NL control and mechanically primed L EVs, both peaking around 150 nm (Figure 2a), consistent with the expected size of exosomes and other small EVs [9, 42]. In addition, scanning electron microscopy (SEM) further verified EV morphology [43], revealing numerous spherical particles smaller than 200 nm (Figure 2b). To examine surface marker composition at the single-EV level, we employed stochastic optical reconstruction microscopy (STORM), a super-resolution microscopy technique, with clustering analysis. This approach identified a heterogenous population of EVs containing one, two, or all three of the canonical tetraspanins, CD9, CD63, and CD81 (Figure 2c–e). The relative distributions of single-, double-, and triple-positive EVs were not notably different between NL and L groups (Figure 2d), indicating that dynamic loading has minimal impact on surface tetraspanin expression. Similarly, the size distributions of these subpopulations were comparable between NL and L groups (Figure 2e), consistent with the NTA results. Taken together, NTA, SEM, and STORM imaging confirm the detected particles to be EVs based on size distributions, morphology, and surface marker expression. In addition to these techniques, transmission electron microscopy (TEM) can provide further ultrastructural details of the EV samples [44]. Overall, these analyses demonstrate that while dynamic mechanical loading markedly increases EV yield, it does not alter their size, morphology, or surface maker composition.

### 2.2 | Dynamic Loading Increases EV Yield through ESCRT-Independent Biogenesis

After establishing that dynamic mechanical loading significantly enhances EV secretion, we next sought to elucidate the mechanisms through which this occurs by identifying the pathways responsible for the observed mechanosensitivity. To this end, several mechanotransduction pathways were pharmacologically inhibited during mechanical loading to identify those contributing to EV production.

Pharmacological inhibition of the Rho/ROCK and actomyosin contractility pathway, which regulates cytoskeletal tension and cell morphology [45], did not affect EV yield under either static free swelling or dynamic loading conditions (Figures 3a and Figure S1). Similarly, blocking the YAP/TAZ transcriptional regulators, key mediators of mechanosensitive gene expression [46], did not alter EV secretion (Figure 3b). Calcium chelation with BAPTA-AM, used to inhibit intracellular calcium flux as a secondary messenger in mechanotransduction [47], also failed to significantly impact EV output (Figure 3c). In contrast, the inhibition of the ESCRT-independent EV biogenesis pathway with GW4869 [48] reduced EV production in non-loading controls, and this reduction could not be fully rescued by dynamic loading (Figure 3d). These results indicate that MFCs predominantly utilize ESCRT-independent mechanisms for EV generation, and that dynamic mechanical loading enhances EV yield, at least in part, through this pathway.

Interestingly, our inhibitor experiments indicated that canonical mechanotransduction pathways, such as Rho/ROCK, YAP/TAZ, and intracellular calcium signaling pathways, did not significantly affect EV production under loading conditions. This contrasts with a prior study showing that flow-induced EV secretion in dental pulp stem cells is mediated by YAP/TAZ signaling [49], suggesting that EV biogenesis is highly dependent on cell type and loading modality. These findings indicate the complexity of EV mechanobiology and suggest that alternative pathways, such as other mechanosensitive Rho GTPases (e.g., RAC1, CDC42) [50] or cell membrane deformation driven by cyclic tensile loading, may contribute to MFC-EV production. In addition, while aligned nanofibrous scaffolds were used to better mimic the circumferentially organized collagen fiber structures of the outer meniscus [1], the extent to which scaffold alignment influences cellular responses to mechanical loading and downstream EV secretion remains unclear and should be further investigated.

### 2.3 | Mechanically Primed EVs Modulate MSC Gene Expression and Cell–Cell Communication

Given that EVs play important roles in cell-to-cell communication and in regulating recipient cell behavior and differentiation [14], and that mechanical loading enhances EV secretion, we next investigated whether mechanically primed MFC-EVs influence recipient MSC behavior. To this end, EVs were collected from MFCs subjected to dynamic tensile loading for either 1 day (1dL) or 3 days (3dL) and subsequently applied to MSC cultures (Figure 4a).

Gene expression analysis of the source MFCs using RT-qPCR revealed an increase in both type I collagen (Col1) and aggrecan (ACAN) expression following 1 h of daily loading for 3 days (Figure 4b). To assess whether EVs secreted under these conditions could impact recipient progenitor cells, MSCs were seeded and treated with the EVs collected from non-loading, 1dL, and 3dL conditions (NL-EVs, 1dL-EVs, and 3dL-EVs, respectively) (Figure 4c). Vybrant CM-DiI-labeled MFC-EVs were observed to be internalized by recipient MSCs within 2 h of treatment initiation (Figure S2), suggesting that cellular uptake likely contributes to their observed effects. CCK-8 assays showed no differences in MSC viability or proliferation after seven days of treatment (Figure 4d), confirming the cytocompatibility of all three EV types. Notably, MSCs treated with mechanically primed EVs exhibited modest gene expression changes suggestive of a fibrocartilage-like profile, including a trend of increased type I collagen (Col1) and significantly elevated aggrecan (ACAN) expression compared to untreated controls (Figure 4e). RT-qPCR assessment of type II collagen (Col2) and transcription factor Sox9 revealed no significant changes with mechanically primed EV treatment (Figure S3). These trends are generally consistent across donors, although some donor heterogeneity was observed (Figure S4).

These data demonstrated that mechanical loading not only increased EV yield but also produced mechanically primed EVs capable of regulating gene expression in recipient MSCs. Specifically, these EVs induced a significant upregulation of aggrecan, a key glycosaminoglycan responsible for the compressive strength of fibrocartilaginous tissues, while maintaining similar trends in type I collagen expression. This pattern closely mirrored that of the source MFCs under applied loading, suggesting that EVs may help transmit load-dependent biochemical signals from MFCs to surrounding progenitor cells. While definitive fibrochondrogenic differentiation cannot be fully assessed at this early time point (7 days), the observed changes in ACAN indicate that mechanically primed MFC-EVs can induce early transcriptional responses associated with matrix production. Further studies incorporating longer culture durations, protein-level analyses, and 3D culture systems will be necessary to determine whether these early changes translate into functional tissue-level outcomes. Together, these findings suggest that EVs derived from mechanically stimulated MFCs may influence MSC gene expression and promote early matrix-associated responses.

### 2.4 | EVs From Dynamically Loaded Constructs Exhibit Distinct Matrix-Associated Protein Cargo Signatures

Given that dynamic mechanical loading influences EV secretion by MFCs, which regulate gene expression behavior in recipient MSCs, we next examined whether mechanical loading altered the protein composition of the mechanically primed EVs. To do this, EV proteins were isolated, biological replicates were pooled, and the samples were analyzed by mass spectrometry following SDS-PAGE separation and stabilization (Figure 5a). Compared with cell-free media controls (NL-neg and 3dL-neg), both NL-EVs and 3dL-EVs displayed distinct and more abundant protein bands (Figure 5a). After subtracting media-derived contaminant proteins, a total of 380 unique proteins were identified in both NL-EVs and 3dL-EVs (Figure 5b). Among these, 48 proteins (12.6% of total) were unique to NL-EVs, 153 proteins (40.3% of total) were unique to 3dL-EVs, and 179 proteins (47.1% of total) were expressed in both pooled samples (Figure 5b). The top 30 proteins in each group accounted for more than 80% of their total protein content (Figure 5c). Representative top 20 proteins in NL-EVs, 3dL-EVs, and those with higher relative abundance in 3dL-EVs relative to NL-EVs (Enriched) are shown in Figure 5d, with a comprehensive list provided in Table S1. The pooled mechanically primed EVs had higher relative abundance of several ECM- and cytoskeleton-associated proteins (Enriched in Figure 5d), including collagens (COL1A1, COL1A2, and COL14) and the small leucine-rich proteoglycan DCN (decorin), reflecting an active ECM remodeling state of the source MFCs. Additionally, several of these proteins have been implicated in pathways relevant to chondrogenesis and matrix production. For example, COL14A1 has been associated with the ERK signaling pathway [51], which plays a role in cellular proliferation and differentiation, as well as extracellular matrix assembly and stabilization [52]. DCN is known to regulate collagen fibrillogenesis, regeneration, and cell migration [53], and has been shown to modulate TGF-*β* signaling, a key pathway in chondrogenesis and proteoglycan synthesis [54]. Other notable EV proteins upregulated by mechanical priming include VIM (vimentin), which has been shown to support MSC-mediated wound closure [55], PCOLCE (procollagen C-endopeptidase enhancer 1), which promotes collagen maturation [56], THBS2 (thrombospondin-2), which is crucial to normal collagen organization and tissue remodeling [57, 58], and TNC (tenascin-C), which is involved in integrin-mediated signaling, inflammatory signaling, and mechanotransduction, which can influence MSC behavior and matrix remodeling [59–62]. Protein-protein interaction analysis demonstrated strong interconnections among the top proteins in NL-EVs, 3dL-EVs, and the most enriched proteins (Figure 5e). Collectively, these findings suggest that dynamic loading is associated with qualitative changes in EV cargo composition, including the preferential representation of ECM-associated proteins. These differences may contribute to the increased ACAN expression observed in recipient MSCs, providing a potential mechanistic connection between mechanical loading, EV cargo, and downstream cellular responses.

Gene Ontology (GO) enrichment of the top 30 proteins in each group showed that both NL-EVs and 3dL-EVs were enriched for biological processes and molecular functions related to negative regulation of catalytic and endopeptidase activity, as well as collagen-associated cellular components (Figures S6 and S7). Notably, proteins with higher relative intensity in 3dL-EVs were enriched for biological processes associated with cell migration, tissue development, and cartilage development (Figure 5f), and for molecular functions involving glycosaminoglycan (GAG) binding (Figure 5h). Cellular components of these proteins were primarily linked to collagen organization and the extracellular matrix (Figure S5).

Together, our proteomic analysis indicates that mechanical priming is associated with a distinct EV cargo composition. 3dL-EVs contained a broader and more diverse protein repertoire than NL-EVs, including preferential representation of proteins with established roles in matrix remodeling and fibrocartilage formation, suggesting that mechanical loading may influence the regenerative potential of MFC-EVs through changes in protein cargo composition. Proteins unique to 3dL-EVs are collectively linked to collagen fibrillogenesis, ECM stabilization, and cytoskeletal remodeling, all of which are key processes for tissue repair. Moreover, GO analysis confirmed that proteins preferentially represented in 3dL-EVs were enriched for biological processes such as cell migration, tissue morphogenesis, and cartilage development, as well as molecular functions related to glycosaminoglycan binding and ECM interactions. Further validation of these proteomic hits using approaches such as Western blotting or immunofluorescence/STORM imaging would strengthen these findings. Repeating the mass spectrometry analysis using independent biological replicates will be necessary to enable differential expression analysis and robust identification of proteins upregulated by mechanical loading. Establishing a causal link between EV protein cargo and cellular responses will require functional assays in recipient cells together with EV perturbation approaches to determine the contribution of specific protein components. Our data suggest that mechanical loading influences both EV yield and cargo composition, with changes consistent with altered matrix remodeling-related biological activity. Together, these findings support the potential relevance of mechanically primed EVs for strategies targeting meniscus repair and tissue homeostasis.

From a translational perspective, our study presents a feasible strategy to overcome one of the primary barriers in EV-based therapeutics: their inherently low yield. Our approach can be scaled up to generate bioactive EV preparations for musculoskeletal repair and regeneration. Dynamic mechanical loading offers a simple and physiologically relevant approach to boost EV production without requiring genetic or chemical manipulation. These findings corroborate other studies that show that modulation of the physical microenvironment can optimize EV output and cargo composition [37]. Importantly, we showed that this strategy may also augment the therapeutic potential of EVs, especially when they are derived from mechanosensitive cell types such as meniscus cells, chondrocytes, or tendon fibroblasts. Previous studies have examined EVs derived from MSCs under mechanical priming conditions, including fluid shear stress [63–65] or dynamic tensile loading [66, 67], and have reported chondroprotective, pro-angiogenic, or anti-inflammatory effects. Our study applies cyclic tensile loading, a physiologically relevant stimulus, directly to outer zone meniscus fibrochondrocytes to modulate MFC-EV production and cargo. To our knowledge, this represents the first study to investigate mechanically primed EVs in a meniscus-specific context and to examine their effects on recipient MSC gene expression.

Despite these advances, there are certain limitations of this study. First, aligned nanofibrous PCL scaffolds were chosen to best represent native physiological conditions for the outer meniscus fibrochondrocytes. However, to further elucidate the role of substrate topology and loading orientation in regulating MFC-EV production, future studies should compare aligned and randomly oriented scaffolds, as well as alternative loading substrates. Such comparisons may provide additional insight into how microenvironmental cues influence mechano-regulated EV secretion and cargo composition. MSC treatment assays were conducted in 2D culture, which is poorly representative of their native environment. Future studies should evaluate EV-mediated effects in 3D scaffold or organoid systems that more closely recapitulate the in vivo microenvironment, enabling a more physiologically relevant assessment of EV function. In addition, extending the study duration will be important to better define the extent of MSC differentiation at both the transcriptional and matrix protein levels, and to more fully assess the functional impact of mechanically primed EVs. Additionally, examining different loading conditions including amplitudes, frequencies, and durations could reveal optimal parameters for maximizing EV yield and regenerative efficacy. Finally, in vivo validation in meniscus injury models will be necessary to confirm the therapeutic potential of mechanically primed EVs and to assess their safety, retention, and bioactivity in joint environments.

## 3 | Conclusions

EVs have emerged as promising tools for regenerative therapies due to their ability to mediate intercellular communication, deliver bioactive cargo, and promote tissue repair. However, their clinical translation remains limited primarily by low production yield and variability in bioactivity. This study demonstrates that physiological mechanical loading of MFCs influences both the biogenesis and cargo composition of MFC-EVs. We demonstrated that dynamic mechanical loading significantly improves MFC-EV quantity through ESCRT-independent mechanisms and simultaneously enriches their cargo with proteins associated with ECM organization and tissue development. These “mechanically primed” EVs may convey load-associated signals that modulate recipient MSC gene expression, suggesting a potential role in load-dependent cell-cell communication within the meniscus environment. Together, our findings provide novel insight into EV-mediated mechanobiology and present a scalable, biologically inspired approach for engineering EV-based strategies to study and potentially modulate musculoskeletal tissue repair.

## 4 | Experimental Section

### 4.1 | Primary Cell Isolation

Fresh juvenile (~3 months) bovine stifle joints (Research 87) were cleaned and the menisci detached from the tibial plateau with a scalpel. MFC isolation was carried out as previously described but only from the outer zone [8, 68]. Briefly, the outer one-third of the tissue was cut from the remaining tissue, chopped into small cubes (2–4 mm), placed sparsely into tissue culture dishes, and covered in basal medium (BM) consisting of Dulbecco’s Modified Eagle’s Medium (DMEM, Gibco) supplemented with 10% fetal bovine serum (FBS, Gibco) and 1% penicillin-streptomycin (Corning). Media were changed every 2–3 days. Cells began migrating out of tissue fragments after 7–10 days of culture, and the remaining tissue fragments were removed after ~14 days. For subsequent use, P0 cells were expanded in BM and either frozen or passaged to P1.

MSCs were isolated from the bovine stifle joint as previously described [69, 70]. Briefly, the femur and tibia were sectioned using a reciprocating saw to expose the trabecular bone. Trabecular bone fragments were removed and submerged in dissection media comprised of DMEM supplemented with 2% penicillin-streptomycin and 1 g heparin/500 mL media. The bone-medium mixture was shaken, and the supernatant was collected into a clean conical tube. The tubes of media were centrifuged, and the resulting pelleted cells were resuspended in BM and plated in tissue culture dishes. Media was changed every 2–3 days. Cells were initially frozen at P0 once they reached 90% confluency. For subsequent use, P0 cells were expanded in BM and either frozen or passaged to P1.

### 4.2 | Preparation of Nanofibrous Scaffolds

Electrospun nanofibrous polycaprolactone (PCL) scaffolds were prepared as described previously [71–73]. Briefly, PCL was dissolved at 35% w/v in a 1:1 (v/v) mixture of N,N-dimethylformamide (DMF) and tetrahydrofuran (THF). A total of 11 mL of PCL solution was loaded into a 20 mL syringe fitted with an 18-guage blunt-tip needle and electrospun onto a rotating and grounded mandrel at a flow rate of 2.5 mL/h. A high voltage of ~17 kV was applied to the solution during electrospinning. To maintain alignment of the collected nanofibers, the surface speed of the mandrel was set at ~10 m/s. The resulting PCL sheet was cut into 21 mm × 55 mm strips with the long side parallel to the prevailing fiber direction. Prior to use PCL scaffolds were rehydrated in decreasing concentrations of ethanol (100%, 70%, 50%, and 30%) for 30 min each, washed in PBS for 30 min, and coated in fibronectin (Sigma) overnight at room temperature. Scaffolds were sterilized for 30 min under UV light prior to cell seeding.

### 4.3 | Bioreactor Loading

Passage 2 MFCs were expanded in tissue culture dishes, trypsinized, and seeded onto scaffolds at a density of ~8 × 10^6^ cells/scaffold in BM. After 2 days of static free swelling culture, scaffolds were transferred to fresh culture dishes containing DMEM supplemented with 10% exosome-depleted FBS (Gibco) and 1% penicillin-streptomycin (Exo-D BM). Loaded scaffolds were affixed into the custom-made bioreactor [70, 74, 75] and subjected to cyclic tensile loading at 3% strain amplitude and 1 Hz frequency. For characterization and inhibitor studies, the scaffolds underwent 3 consecutive hours of loading. For 1-day and 3-day loading experiments, each round of loading consisted of 1 h of loading followed by 3 h of free swelling. In the 3-day loading groups, scaffolds were removed from the bioreactor and returned to the same culture medium overnight between rounds, for a total of three loading cycles (Figure 4a). Static (NL) controls remained in free-swelling conditions for the duration of the loading period. At the completion of loading, culture medium was collected for further processing. For RNA extraction, the central region of each scaffold was cut with a scalpel into small (~2–3 mm) squares and placed into TRIzol reagent for subsequent processing.

### 4.4 | EV Isolation and Characterization

Culture medium collected from scaffolds was first centrifuged at 500 × g for 5 min at 4°C. The supernatant was transferred to a clean tube and centrifuged again at 2000 × g for 20 min at 4°C. The resulting supernatant was transferred to 38.5 mL ultra-clear open-top tubes (Beckman) and ultracentrifuged twice at 100 000 × g (24 200 rpm) for 70 min at 4°C using an SW Ti 32 rotor in a Beckman Coulter Optima XPN-80 Ultracentrifuge. After each ultracentrifugation step, the supernatant was carefully removed using serological and micropipettes. The final EV pellet was resuspended in ~200–500 μL of PBS.

Isolated EVs were characterized by nanoparticle tracking analysis (NTA) using the ZetaView PMX220 Twin. Briefly, resuspended EVs were further diluted in 2 mL of PBS to achieve a density of particles on the order of 10^5–^10^10^ particles per milliliter. 1 mL of the diluted sample was injected into the cell assembly at 25°C. The instrument then recorded a video from which the particles’ Brownian motion was analyzed to determine their size and concentration. Total EV quantities were calculated using these particle concentrations and the volume of the initial resuspension after ultracentrifugation.

EV morphology was confirmed by scanning electron microscopy (SEM). Briefly, EV suspensions were mixed with an equal volume of 8% glutaraldehyde and incubated overnight at 4°C to fix. The EVs were then transferred into a 100 kDa centrifugal filter tube, washed with sterile PBS, and centrifuged at 2000 × g for 3 min. Following this, the EVs were dehydrated with increasing concentrations of ethanol (80%, 90%, and 100%). For each ethanol concentration, approximately 10–15 mL of ethanol was added to the filter tube and centrifuged at 2000 × g for 3 min and the filtrate was discarded after each step. The dehydrated samples were resuspended to a final volume of 1 mL and transferred to a clean cover glass to dry. The EVs were sputter coated with iridium using an EMS/Quorum Q150T ES sputter coater and imaged on the FEI Quanta FEG 250 scanning electron microscope.

Stochastic Optical Reconstruction Microscopy (STORM) was performed using the Oxford Nanoimager to visualize EV size and distribution of surface markers CD9, CD63, and CD81 (ONI). Samples were prepared according to the EV Profiler Kit 2 manufacturer’s instructions and co-stained with the antibodies prior to imaging. Images were acquired and analyzed using the AutoEV and CODI platforms.

### 4.5 | Inhibitor Treatments

To assess the effects of pathway-specific inhibitors on EV production, scaffolds were prepared and seeded as above. To inhibit the Rho/ROCK signaling pathway, Y27632 (10 μM) was added to scaffolds in Exo-D BM for 1 h prior to loading [46]. To inhibit actomyosin contractility, blebbistatin (50 μM) was added to scaffolds in Exo-D BM for 1 h prior to loading [46]. To inhibit YAP/TAZ signaling, verteporfin (1 μM) was added to scaffolds in Exo-D BM immediately prior to loading [49]. To inhibit calcium signaling, calcium chelator BAPTA-AM (25 μM) was added to scaffolds in Exo-D BM for 30 min prior to loading [76]. To inhibit ESCRT-independent EV biogenesis, GW4869 (20 μM) was added to scaffolds in Exo-D BM 24 h prior to loading [38]. All inhibitor studies were conducted using the same loading conditions of 3 h of cyclic tensile loading at 3% strain amplitude and 1 Hz frequency. All respective static controls were maintained in identical media conditions containing the respective inhibitors for the same durations as the loaded groups. All inhibitors were purchased from Sigma.

### 4.6 | EV Treatment of MSCs

MSCs were expanded in 15 cm tissue culture dishes, then seeded into 12-well tissue culture plates at a density of 2 × 10^5^ cells/well in BM. 1 day after seeding, the media was replaced with a chemically defined medium (CM-) consisting of DMEM, dexamethasone (100 μM), ascorbate-2-phosphate (50 μg/mL), L-proline (40 μg/mL), sodium pyruvate (100 μg/mL), ITS+ premix (1X, Corning), penicillin-streptomycin (1%), bovine serum albumin (1.25 mg/mL), and linoleic acid (5.3 μg/mL). For EV treatments, 1 × 10^4^ EVs/cell (based on the cell seeding density) was added to each well (Figure 4c). Isolated EVs were labeled with Vybrant CM-DiI (Invitrogen) according to manufacturer’s instructions. To remove unbound dye, labeled EVs were washed with PBS and subjected to a final round of ultracentrifugation as previously described. At the indicated time points, treated cells were fixed with 4% paraformaldehyde. Cell cytoskeletal F-actin was stained using phalloidin-Alexa488 (Invitrogen) for 1 h at 37°C. Cell nuclei were stained using Hoechst 33342 (Invitrogen) per manufacturer’s instructions. Fluorescent uptake images were obtained using a Leica DM6000 widefield microscope. Cell viability was assessed using the CCK-8 reagent according to the manufacturer’s protocol. For gene expression analysis, EV treatments were carried out over 7 days, with an EV-containing media change on day 3. On 7 days, the cells were lysed in TRIzol reagent for RNA extraction.

### 4.7 | Gene Expression Analysis with Real-Time Quantitative PCR

Scaffolds or cells in TRIzol underwent RNA extraction using Zymo RNA mini-prep columns (Zymo Research, Irvine, CA), according to the manufacturer’s protocols. Extracted RNA was converted to cDNA using the ProtoScript II Reverse Transcriptase kit (NEB) using random primers according to manufacturer’s protocols. Real-time quantitative PCR (RT-qPCR) was carried out using SYBR Green Master Mix (Applied Biosystems) according to manufacturer’s protocols. Primer sequences for RT-qPCR are listed in Table 1. GAPDH was used as the housekeeping reference gene, and relative gene expression levels were calculated using the ΔΔCT method.

### 4.8 | Proteomic Analyses

For proteomic analysis, EVs were isolated using polyethylene glycol (PEG) precipitation as previously described [77]. To prepare PEG solution, 10 kDa PEG was reconstituted in deionized water at a 50% w/v ratio until a homogenous solution was achieved. The solution was sterile-filtered through a 0.22 um filter and used as the stock solution. Cell culture media was collected from both cell-free (negative controls) and cell-containing scaffolds, which were seeded and loaded either once or over three days as described. The media was centrifuged at 500 × g for 5 min followed by 2000 × g for 20 min to clear cell debris. Then, 8 mL of each media sample was mixed with 2 mL of PEG stock solution and incubated at 4°C overnight. The following day, the samples were centrifuged at 3000 × g for 10 min at 4°C. The supernatant was carefully decanted and aspirated from the pellet, which was then resuspended in 50 μL RIPA buffer and vortexed to lyse proteins. Protein concentrations were analyzed using bicinchoninic acid (BCA) protein assay (Thermo) according to manufacturer’s protocols. Equal amounts of protein (18 μg) per sample were prepared in 4x Laemmli reducing sample buffer, boiled for 5 min at 105°C for protein denaturation, and run on a precast 4%–20% polyacrylamide gel (Bio-Rad). For SDS-PAGE analysis, samples were run in adjacent wells at 100 V for approximately 1 h. Gels were then rinsed with deionized water, stained with Coomassie blue for 1 h at room temperature, washed with deionized water, and destained overnight. Protein bands were visualized using a Gel Doc imaging system.

For mass spectrometry, samples were run with a minimum of 3 wells of separation to minimize crossover contamination at 100 V for approximately 30 min. Protein bands were then fixed in a solution of methanol and acetic acid. The fixed gels were stained with Coomassie blue at 4°C for 1 day and subsequently destained for 1 day. After destaining, the protein bands were carefully cut from the gel and placed into a 2 mL microcentrifuge tube filled with deionized water. Samples were then shipped to the Taplin Mass Spectrometry Facility at Harvard University for liquid chromatography-tandem mass spectrometry (LC-MS/MS). Media proteins found in the static and mechanically primed negative controls were subtracted from the cell-containing samples by their intensity percentages. Protein networks of the top 20 expressed and upregulated proteins were visualized and annotated using the Cytoscape tool (version 3.10.4, Cytoscape Consortium 2025) [78]. Gene ontology (GO) enrichment analyses were conducted using the STRING web tool (version 12.0, http://string-db.org, String Consortium 2025) to identify biological process, molecular function, and cellular component terms with a false discovery rate (FDR) <0.05.

### 4.9 | Statistical Analysis

All numerical data are reported as the mean and standard deviation of biological replicates. Three to four biological replicates were used in all inhibitor and cell treatment studies. For EV isolation, four technical replicates from separate wells were pooled for downstream processes (Figure 1a). For mass spectrometry, EVs or media were collected from four biological donors and pooled before running SDS-PAGE. Student’s *t*-tests or one-way analysis of variance (ANOVA) with Tukey’s *post hoc* testing, as applicable, were performed on quantified data. Statistical significance was reached at p<0.05. Outliers in numerical datasets were determined using Grubbs’ test with a significance level of alpha = 0.05. All statistical analyses were carried out on GraphPad Prism 10.

## Supplementary Material

Suppl Figure 1

Suppl Figure 3

Suppl Figure 4

Suppl Figure 2

Suppl Figure 5

Suppl Figure 6

Suppl Figure 7

Suppl Table 1

Supporting Information

Additional supporting information can be found online in the Supporting Information section.

**Supporting File**: admi70586-sup-0001-SuppMat.docx.

## Figures and Tables

**FIGURE 1 |:**
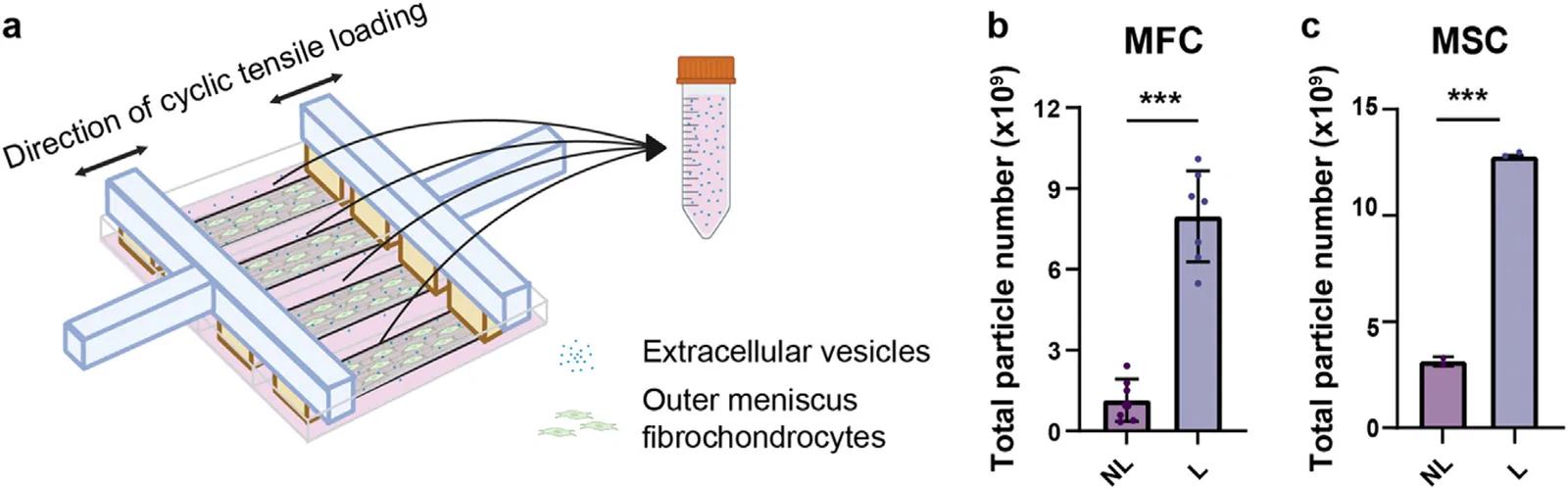
Dynamic mechanical loading increases EV yield in multiple cell types. (a) Schematic of the custom bioreactor setup and EV collection process; medium is collected from four technical replicate samples per condition and pooled for subsequent EV isolation and analysis. Illustration created with BioRender (https://BioRender.com/5z9hfxz). (b) Comparison of EV yield between dynamic loading (L) and non-loading (NL) MFCs (n=7 biological replicates, *** *p* <0.001). (c) Comparison of EV yield between L and NL MSCs (n=3 biological replicates, *** *p* <0.001).

**FIGURE 2 |:**
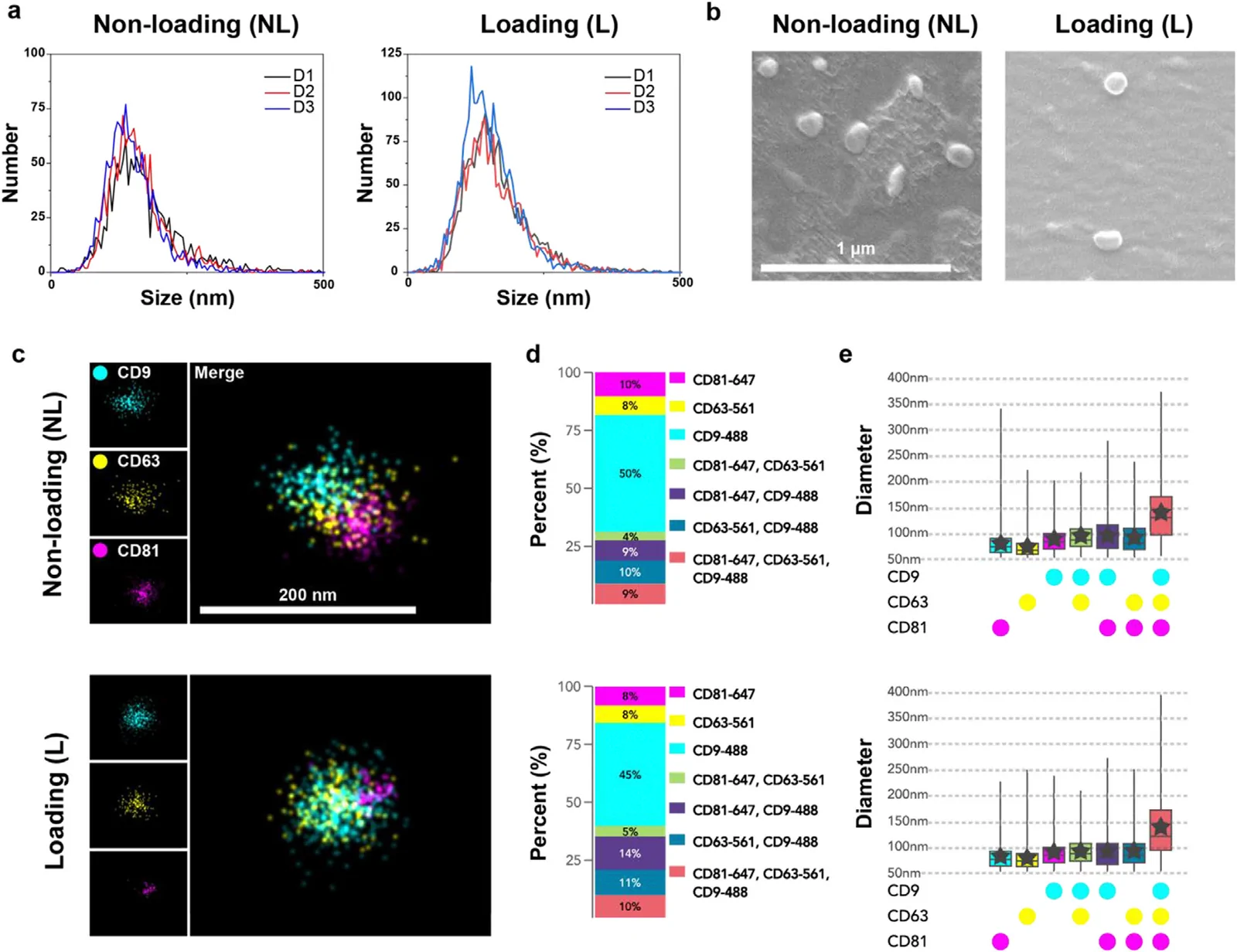
Characterization of isolated MFC-EVs. (a) Representative size distributions of collected EVs analyzed by nanoparticle tracking analysis (NTA) from 3 different donors (D1–D3). Representative images acquired using (b) scanning electron microscopy (SEM) and (c) super-resolution microscopy (STORM) of NL-EVs and L-EVs. (d) Quantification of STORM clustering analysis showing proportions of EVs positive for individual (CD9, CD63, or CD81), dual, or triple tetraspanin expression. (e) Size distributions of collected EVs using STORM imaging and clustering analysis. (NL: n=2144 EVs, L: n=2326 EVs).

**FIGURE 3 |:**
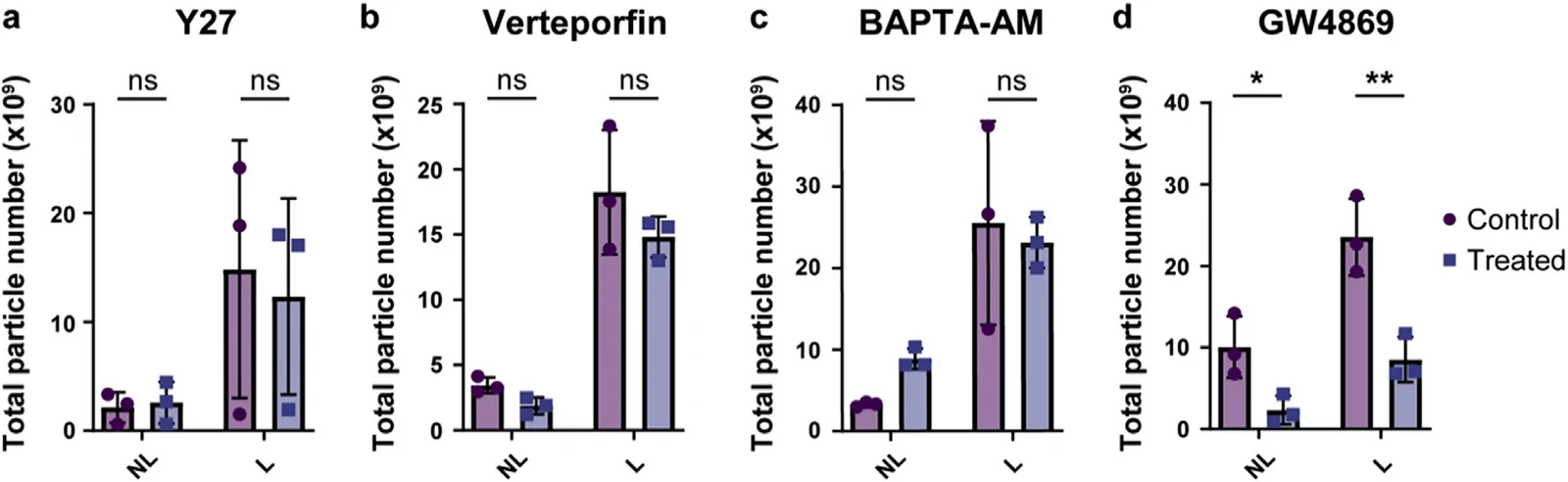
Identification of mechanoresponsive pathways regulating EV secretion in MFCs. Total EV particles quantified by nanoparticle tracking analysis (NTA) under non-loading (NL) and dynamic loading (L) conditions following treatment with pharmacological inhibitors: (a) Y27632 (Rho/ROCK inhibitor), (b) Verteporfin (YAP/TAZ inhibitor), (c) BAPTA-AM (calcium chelator), and (d) GW4869 (inhibitor of ESCRT-independent EV biogenesis pathway). All inhibitor studies were conducted using n=3 biological donors, Student’s *t*-test, * *p* <0.05, ** *p* <0.01.

**FIGURE 4 |:**
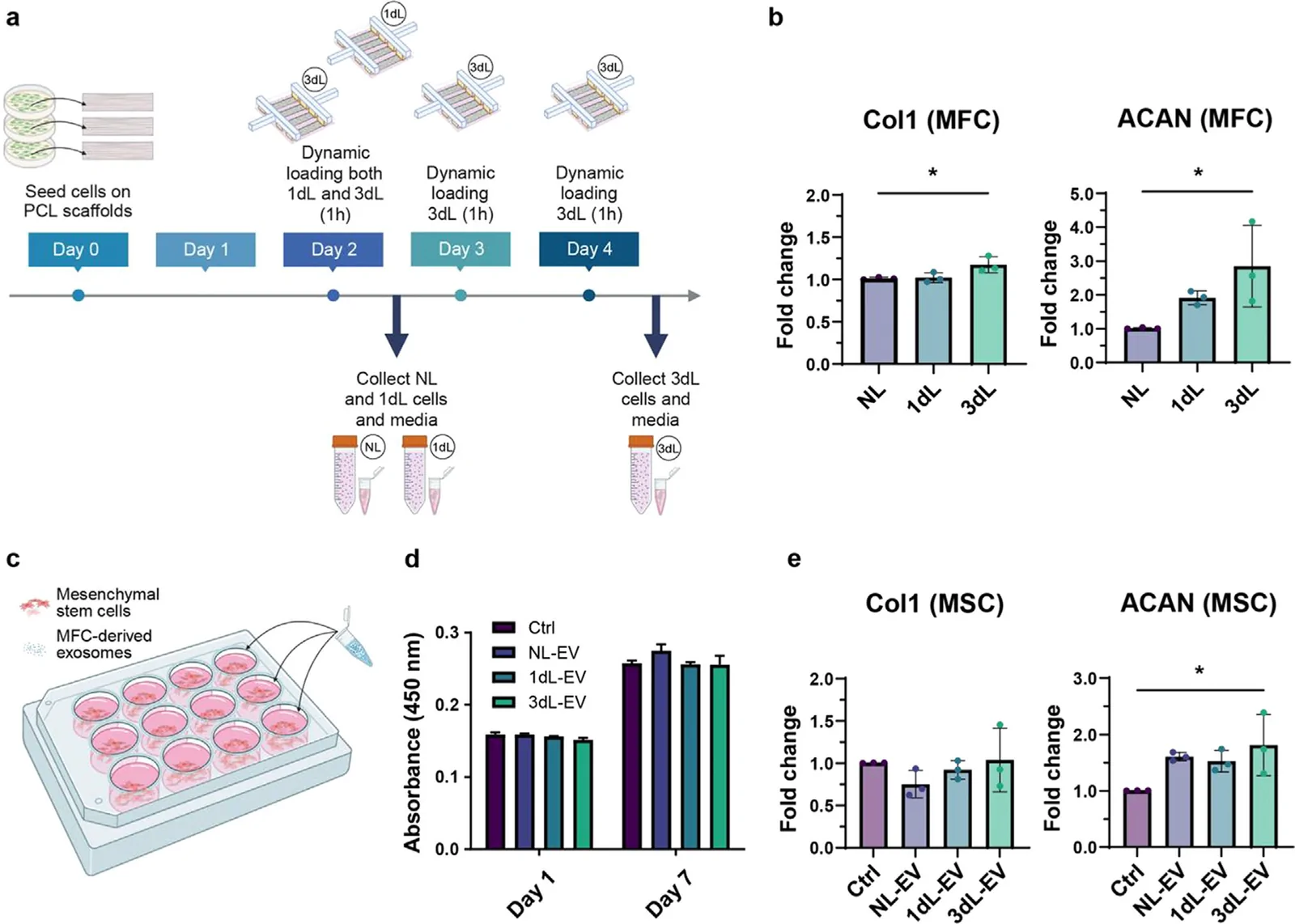
Mechanically primed MFC-EVs regulate recipient MSC gene expression. (a) Schematic of loading regimen: MFCs were subjected to dynamic cyclic tensile loading for 1 h either once (1dL) or daily for three consecutive days (3dL). Non-loading (NL) MFCs were maintained under free-swelling conditions for the equivalent duration. Illustration was created with BioRender (https://BioRender.com/es3u9z1). (b) Gene expression analysis of NL, 1dL, and 3dL MFCs (n=3 biological replicates, one-way ANOVA, * *p* <0.05). (c) Schematic of EV treatment design: MSCs were treated with donor-matched MFC-EVs for 7 days. (d) Cell viability and proliferation assays of EV-treated MSCs (n=3 biological replicates). (e) Gene expression analysis of MSCs after 7 days of treatment with NL-, 1dL-, or 3dL-EVs (n=3 biological replicates, one-way ANOVA, * *p* <0.05).

**FIGURE 5 |:**
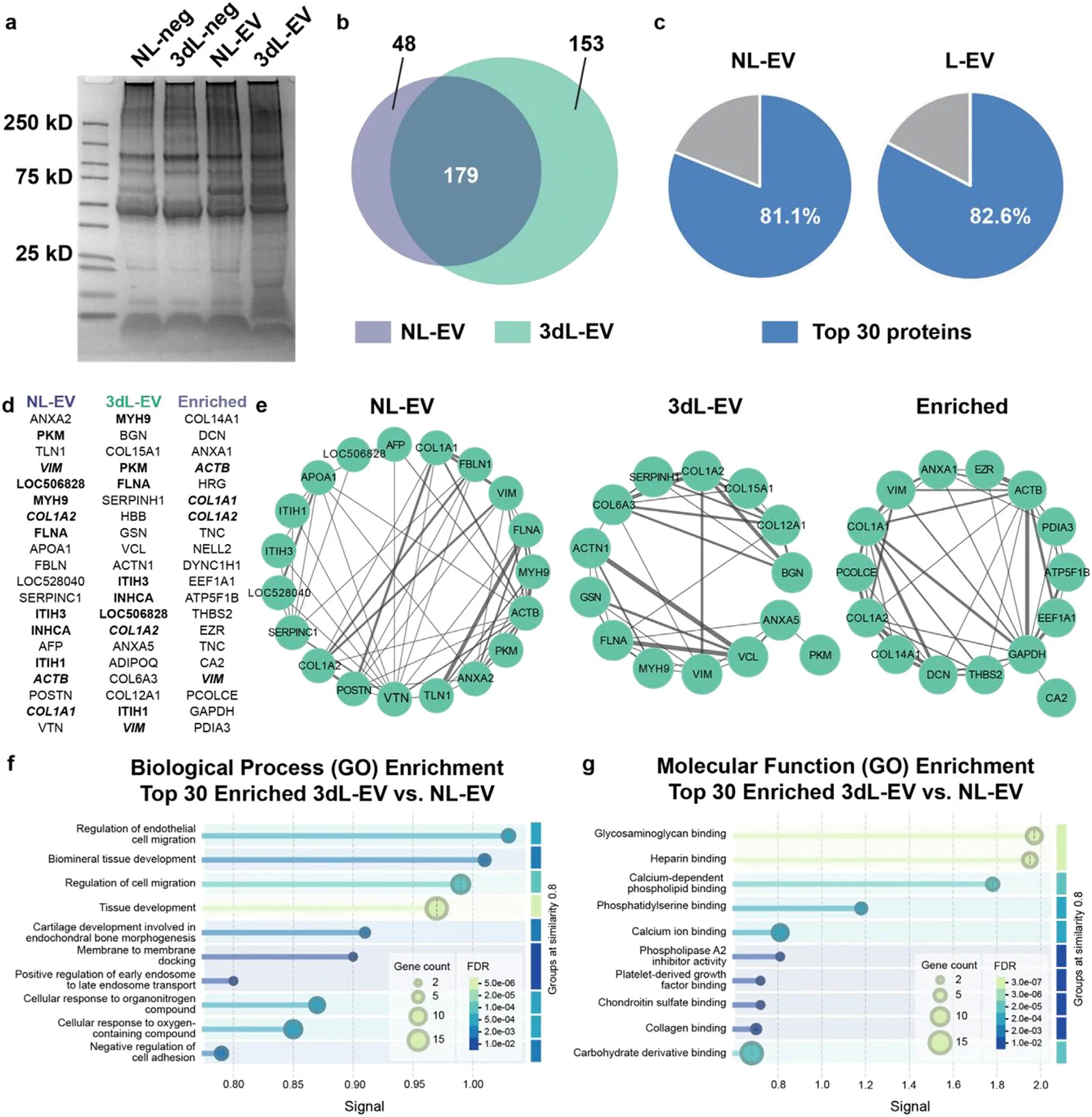
Proteomic profiling of MFC-derived EVs using liquid chromatography-tandem mass spectrometry (LC-MS/MS). (a) SDS–PAGE analysis of samples prepared for proteomics compared with a Bio-Rad protein ladder. Groups (left → right): non-loading without cells (NL-neg), loading without cells (3dL-neg), non-loading with cells (NL-EV), and loading with cells (3dL-EV). (b) Venn diagram showing overlapping and unique proteins identified in NL- and 3dL-EVs. (c) Relative proportion of the top 30 proteins in NL-EVs (left) and 3dL-EVs (right). (d) Top 20 proteins detected in NL-EVs, 3dL-EVs, and those with higher relative abundance in 3dL-EVs compared with NL-EVs (Enriched). Bold indicates proteins shared between NL- and 3dL-EVs; bold-italics indicate proteins also found among the top enriched proteins. (e) Protein–protein interaction (PPI) networks of the top proteins in NL-EVs, 3dL-EVs, and the most enriched proteins in 3dL-EVs. (f) Gene Ontology (GO) enrichment of the top 30 enriched proteins highlighting biological processes, and (h) molecular functions, where dot size and color represent gene counts and false-discovery rates (FDRs), respectively.

**TABLE 1 |:** Forward and reverse primer sequences used for quantitative real-time PCR analysis.

Gene	Forward (5’ → 3’)	Reverse (5’ → 3’)

ACAN	CCTGAACGACAAGACCATCGA	TGGCAAAGAAGTTGTCAGGCT
COL1A2	AATTCCAAGGCCAAGAAGCATG	GGTAGCCATTTCCTTGGTGGTT
COL2	GTGTCAGGGCCAGGATGTC	GCAGAGGACAGTCCCAGTGT
SOX9	TGAAGAAGGAGAGCGAGGAG	GTCCAGTCGTAGCCCTTGAG

## Data Availability

The data that support the findings of this study are available from the corresponding author upon reasonable request.
