# Supplementary material for: Dynamic Mechanical Loading Modulates Extracellular Vesicle Production and Cargo in Meniscus Fibrochondrocytes
Source: Adv Mater Interfaces. Author manuscript; Available in PMC 2026 Nov 13. (PMC13756810; doi:10.1002/admi.202600012)
Supplement: Supplementary file 1 [file NIHMS2212607-supplement-Suppl_Figure_1.docx]

**Supplementary Figures and Legends**

**Figure S1:** Total EV yield from non-loading (NL) and dynamic loading (L) MFCs treated with blebbistatin(−), an inhibitor of actomyosin contractility (n=2 biological replicates, Student’s t-test, ns: p>0.05)
