# Supplementary material for: Dynamic Mechanical Loading Modulates Extracellular Vesicle Production and Cargo in Meniscus Fibrochondrocytes
Source: Adv Mater Interfaces. Author manuscript; Available in PMC 2026 Nov 13. (PMC13756810; doi:10.1002/admi.202600012)
Supplement: Supplementary file 2 [file NIHMS2212607-supplement-Suppl_Figure_2.docx]

**Supplementary Figures and Legends**

**Figure S2:** Uptake of Vybrant CM-DiI-labeled EVs by recipient MSCs. Cells were fixed, stained, and imaged at (a) 30 minutes, (b) 1 hour, and (c) 2 hours after treatment. Scale bar = 20 µm.
