# Supplementary material for: Dynamic Mechanical Loading Modulates Extracellular Vesicle Production and Cargo in Meniscus Fibrochondrocytes
Source: Adv Mater Interfaces. Author manuscript; Available in PMC 2026 Nov 13. (PMC13756810; doi:10.1002/admi.202600012)
Supplement: Supplementary file 3 [file NIHMS2212607-supplement-Suppl_Figure_3.docx]

**Supplementary Figures and Legends**

**Figure S3:** Type II collagen (Col2) and Sox9 gene expression by MSCs treated with mechanically primed MFC-EVs, assessed by RT-qPCR. (n=3 biological replicates, one-way ANOVA).
