# Supplementary material for: Dynamic Mechanical Loading Modulates Extracellular Vesicle Production and Cargo in Meniscus Fibrochondrocytes
Source: Adv Mater Interfaces. Author manuscript; Available in PMC 2026 Nov 13. (PMC13756810; doi:10.1002/admi.202600012)
Supplement: Supplementary file 4 [file NIHMS2212607-supplement-Suppl_Figure_4.docx]

**Supplementary Figures and Legends**

**Figure S4:** Types I and II collagen (Col1, Col2), aggrecan (ACAN), and Sox9 gene expression of MSCs treated with mechanically primed MFC-EVs, by donor. Means represent three to four technical replicates, error bars represent SD of technical replicates.
