# Supplementary material for: Dynamic Mechanical Loading Modulates Extracellular Vesicle Production and Cargo in Meniscus Fibrochondrocytes
Source: Adv Mater Interfaces. Author manuscript; Available in PMC 2026 Nov 13. (PMC13756810; doi:10.1002/admi.202600012)
Supplement: Supplementary file 5 [file NIHMS2212607-supplement-Suppl_Figure_5.docx]

**Supplementary Figures and Legends**

**Figure S5:** Gene Ontology (GO) enrichment analysis of the top 30 upregulated proteins in dynamic mechanical loading (3dL) EVs compared to non-loading (NL) EVs.
