# Supplementary material for: Dynamic Mechanical Loading Modulates Extracellular Vesicle Production and Cargo in Meniscus Fibrochondrocytes
Source: Adv Mater Interfaces. Author manuscript; Available in PMC 2026 Nov 13. (PMC13756810; doi:10.1002/admi.202600012)
Supplement: Supplementary file 6 [file NIHMS2212607-supplement-Suppl_Figure_6.docx]

**Supplementary Figures and Legends**

**Figure S6:** Gene Ontology (GO) enrichment analyses of the top 30 proteins identified in non-loading (NL) EVs. Analyses include (a) biological processes, (b) molecular functions, and (c) cellular components.
